# Does Bowel Preparation for Colonoscopy Affect Cognitive Function?

**DOI:** 10.1097/MD.0000000000001823

**Published:** 2015-11-06

**Authors:** P. Wadsworth, H. Blackburne, L. Dixon, B. Dobbs, T. Eglinton, A. Ing, R. Mulder, R.J. Porter, C. Wakeman, F.A. Frizelle

**Affiliations:** From the Colorectal Unit, Department of Surgery, Christchurch Hospital, Christchurch, New Zealand (PW, HB, LD, BD, TE, AI, RM, RJP, CW, FAF) and Department of Psychological Medicine, University of Otago, Christchurch (RM and RP).

## Abstract

Colonoscopy is a common procedure used in the diagnosis and treatment of a range of bowel disorders. Prior preparation involving potent laxatives is a necessary stage to ensure adequate visualization of the bowel wall. It is known that the sedatives given to most patients during the colonoscopy cause a temporary impairment in cognitive function; however, the potential for bowel preparation to affect cognitive function has not previously been investigated. To assess the effect of bowel preparation for colonoscopy on cognitive function. This was a prospective, nonrandomized controlled study of cognitive function in patients who had bowel preparation for colonoscopy compared with those having gastroscopy and therefore no bowel preparation. Cognitive function was assessed using the Modified Mini Mental State Examination (MMMSE) and selected tests from the Cambridge Neuropsychological Test Automated Battery. Individual test scores and changes between initial and subsequent tests were compared between the groups. Age, gender, and weight were also compared. Forty-three colonoscopy and 25 gastroscopy patients were recruited. The 2 groups were similar for age and gender; however, patients having gastroscopy were heavier. MMMSE scores for colonoscopy and gastroscopy groups, respectively, were 28.6 and 29.5 (*P* = 0.24) at baseline, 28.7 and 29.8 (*P* = 0.32) at test 2, 28.1 and 28.5 (*P* = 0.76) at test 3. Motor screening scores for colonoscopy and gastroscopy groups, respectively, were 349.3 and 354.1 (*P* = 0.97) at baseline, 307.5 and 199.7 (*P* = 0.06) at test 2, 212.0 and 183.2 (*P* = 0.33) at test 3. Spatial working memory scores for colonoscopy and gastroscopy groups, respectively, were 14.4 and 6.7 (*P* = 0.29) at baseline, 9.7 and 4.3 (*P* = 0.27) at test 2, 10 and 4.5 (*P* = 0.33) at test 3. Digit Symbol Substitution Test scores for colonoscopy and gastroscopy groups, respectively, were 36.3 and 37.8 (*P* = 0.84) at baseline, 36.4 and 40.0 (*P* = 0.59) at test 2, 38.6 and 40.8 (*P* = 0.76) at test 3.

This study did not find evidence of cognitive impairment resulting from administration of bowel preparation before colonoscopy.

## INTRODUCTION

Colonoscopy is a common procedure used in the diagnosis and treatment of a range of bowel disorders. Prior preparation involving potent laxatives is a necessary stage to ensure adequate visualization of the bowel wall. This is usually undertaken by the patient at home within the 24 to 48 hours preprocedure, they are then expected to make their own arrangements to travel to hospital on the day of the procedure. Consent for the colonoscopy is usually taken and/or confirmed immediately before the procedure.

It is known that the sedatives given to most patients during the colonoscopy cause a temporary impairment in cognitive function^1^ and that it can be unsafe to discharge patient's unaccompanied following sedation.^2^ Patients are also advised not to drive or make any significant legal or financial decisions for 24 hours after sedation. Adverse events relating to bowel preparation, such as seizures or unconsciousness secondary to electrolyte imbalance, have been reported.^3,4^ However, the potential for bowel preparation to affect cognitive function has not previously been investigated. If such an effect was found it would be appropriate to advise patients of this in the same way as for patients undergoing sedation so that they could modify their activities accordingly. The capacity of patients to consent to colonoscopy after undergoing bowel preparation would also be brought into question.

However, the potential for bowel preparation to affect cognitive function has not previously been investigated as assessed by the Modified Mini Mental State Examination (MMMSE) and Cambridge neuropsychological testing (CANTAB).

## METHOD

This was a prospective, nonrandomized, study of cognitive function in patients having bowel preparation for colonoscopy compared with patients having gastroscopy who had no bowel preparation. Ethical approval was granted by the regional ethics committee.

All endoscopy providers at Christchurch Hospital were approached for permission to involve their patients in the study; data collection ran over a 2-year period from August 2010. Patients were included if they were booked for outpatient gastroscopy or colonoscopy and their address was deemed to be within 20 km traveling distance of Christchurch Hospital. To allow easy travel times for assessment of cognitive function. Patients were excluded if they were rest home residents, under 18, had known learning difficulties or cognitive impairment, these having gastroscopy and colonoscopy, and those attending for bowel preparation as inpatients were also excluded. The standard agent used for bowel preparation at Christchurch Hospital at the time of the study was Picosulfate (Picoprep®). Suitable patients received letters or phone calls inviting them to participate up to 1 to 2 weeks before the date of their endoscopy.

**FIGURE 1:**
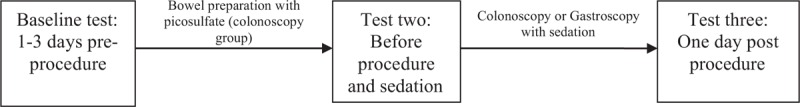
The sequence of testing in the study.

Patients were tested on 3 occasions, each time in the same order by the same administrator (LD). Baseline testing was performed 1 to 3 days before preparation or procedure, test 2 was performed on the day of the procedure before sedation, and the final test was performed on the day after the procedure (Figure 1). Tests used were MMMSE^5^ and selected CANTAB^6^ tasks: motor screening (MSL) which measures latency of response when participants touch a cross appearing on a computer screen therefore lower scores represent better performance, and spatial working memory (SWM) which involves searching “boxes” for a “token” without returning to previously explored boxes, with accuracy and latency being combined to give an overall performance score, where lower score represents better performance. In addition participants performed the following tasks. Digit Symbol Substitution Test (DSST) requires the participant to record the correct numbers under a set of symbols based on predefined pairs with number of correct pairs in 90 seconds recorded, meaning higher score represents better performance. Digit span involves memorizing a series of digit spans, increasing in length from 3 to 9 numbers. Trial 1 (digit span forwards) requires the participant to repeat the numbers back in order. In the second trial (digit span backwards) the subject is asked to repeat each span of digits in reverse order. The number of spans correct is tallied for a total score in each component and an overall score therefore higher scores represent improved performance.

Immediately before the procedure an intravenous cannula for medications such as sedation and analgesia was inserted; 5 ml of blood was drawn from this before any medication was administered for testing of urea and electrolytes

Data was analyzed in SPSS for Windows using repeated measures analysis of variance; multivariate analysis of variance (MANOVA) with test occasion as a within subject variable and group (colonoscopy/gastroscopy) as a between subjects variable. Kruskal-Wallis independent samples test was used to compare groups at the same time point; related samples Wilcoxon rank test was used to compare performance of each individual group at the 3 time points.

## RESULTS

Seventy-two patients were recruited for the study. Adequate data were collected on 68; 43 for colonoscopy and 25 for gastroscopy. The reasons for noninclusion of data were that 1 person was unable to attend for colonoscopy due to heavy snow, and 3 patients results could not be included in the analysis due to computer errors. The study population demographics are shown in Table 1. As shown there were no significant differences between groups except in weight with gastroscopy patients being significantly heavier on average. Three subjects who underwent both colonoscopy and gastroscopy were analyzed in the colonoscopy group.

**TABLE 1:**
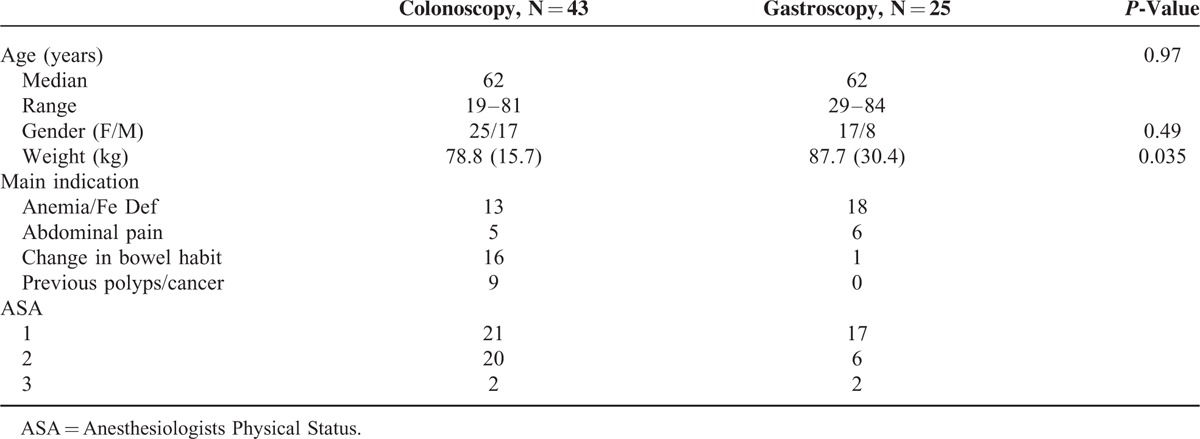
Description of Study Population

Visual inspection of residual plots was used to determine significant departure from a normal distribution of data and revealed such a departure only for the results of the MMSE. These data were therefore analyzed by nonparametric methods. Kruskal-Wallis independent samples test was used to compare groups at the same time point. Related samples Wilcoxon rank test was used to compare performance of each individual group at the 3 time points.

Median MSE score at baseline was 29 for all patients with no significant difference between groups (*P* > 0.1). Median MSE score increased to 30 at Time 1 and Time 2. There was a significant improvement in scores in the colonoscopy group from Baseline to Time 1 and from Baseline to Time 2 (*P* < 0.01). The difference from Time 2 to Time 3 was not statistically significant. There were no significant differences between time points in the gastroscopy group. No patient scored below 24 points at any time point (Table 2).

**TABLE 2:**
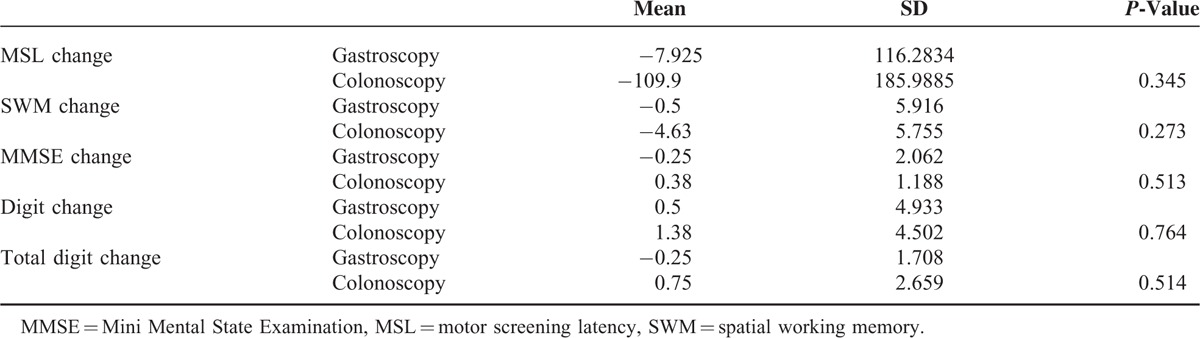
Changes Between Tests in Sessions 1 and 2

For all other tests MANOVA results are shown in Table 3 together with residual mean scores. For SWM between search errors the significant effect of time represents an improvement in performance with each subsequent test, in both groups.

**TABLE 3:**
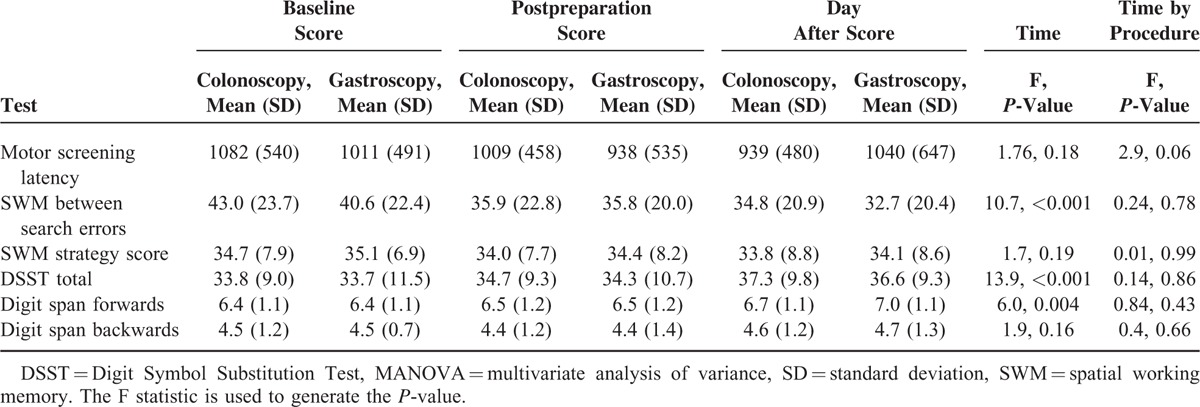
Effects of Bowel Preparation on Cognitive Function in Colonoscopy Patients Compared With No Preparation in Gastroscopy Patients

**FIGURE 2:**
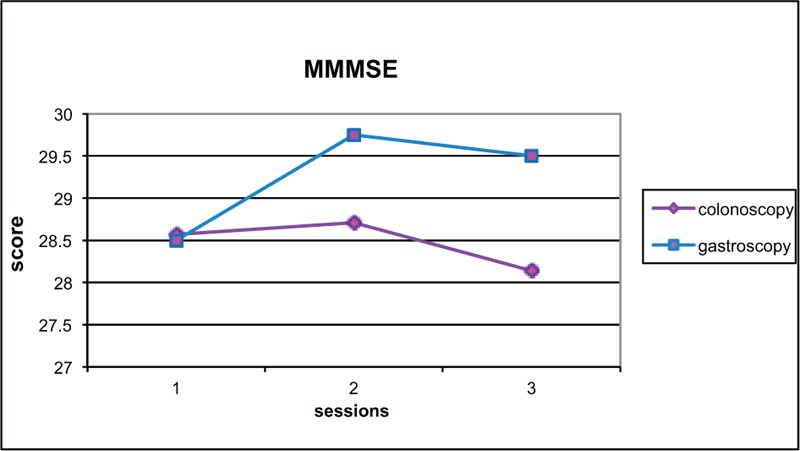
Modified Mini Mental State Examination (MMMSE) scores for colonoscopy and gastroscopy groups, respectively, were 28.6 and 29.5 (no significant difference *P* = 0.24) at baseline, 28.7 and 29.8 (no significant difference *P* = 0.32) at test 2, 28.1 and 28.5 (no significant difference *P* = 0.76) at test 3.

**FIGURE 3:**
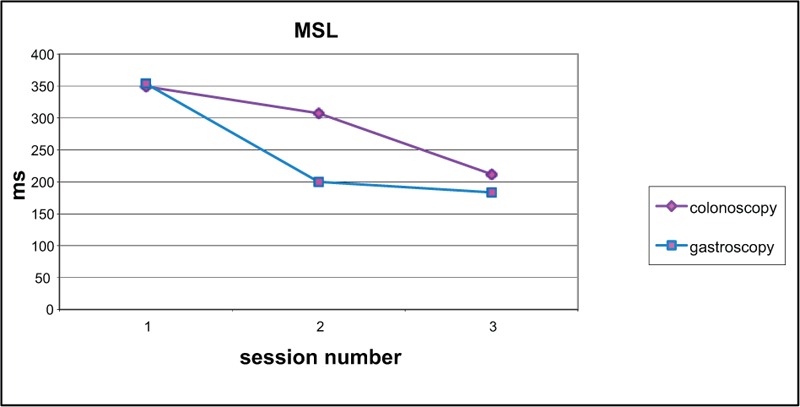
Motor screening (MSL) scores for colonoscopy and gastroscopy groups, respectively, were 349.3 and 354.1 (no significant difference *P* = 0.97) at baseline, 307.5 and 199.7 (no significant difference *P* = 0.06) at test 2, 212.0 and 183.2 (no significant difference *P* = 0.33) at test 3.

**FIGURE 4:**
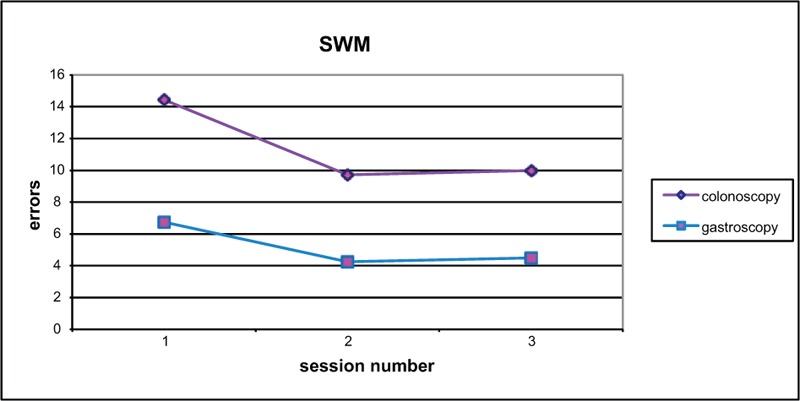
Spatial working memory (SWM) scores for colonoscopy and gastroscopy groups, respectively, were 14.4 and 6.7 (no significant difference *P* = 0.29) at baseline, 9.7 and 4.3 (no significant difference *P* = 0.27) at test 2, 10 and 4.5 (no significant difference *P* = 0.33) at test 3.

**FIGURE 5:**
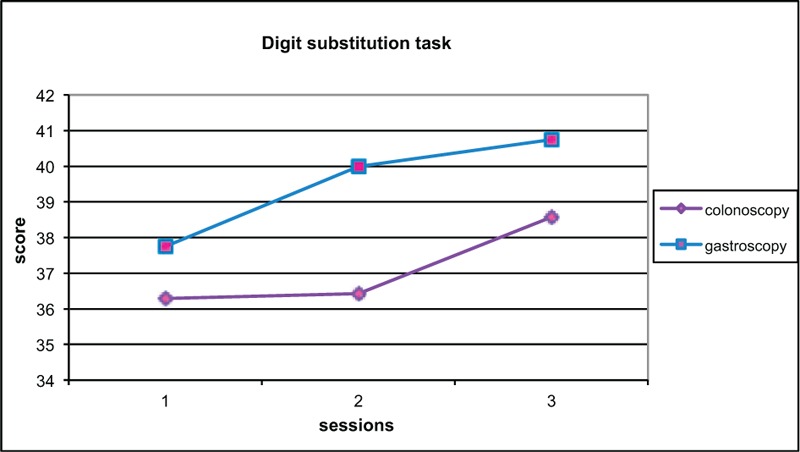
Overall Digit Symbol Substitution Test (DSST) scores for colonoscopy and gastroscopy groups, respectively, were 36.3 and 37.8 (no significant difference *P* = 0.84) at baseline, 36.4 and 40.0 (no significant difference *P* = 0.59) at test 2, 38.6 and 40.8 (no significant difference *P* = 0.76) at test 3.

**FIGURE 6:**
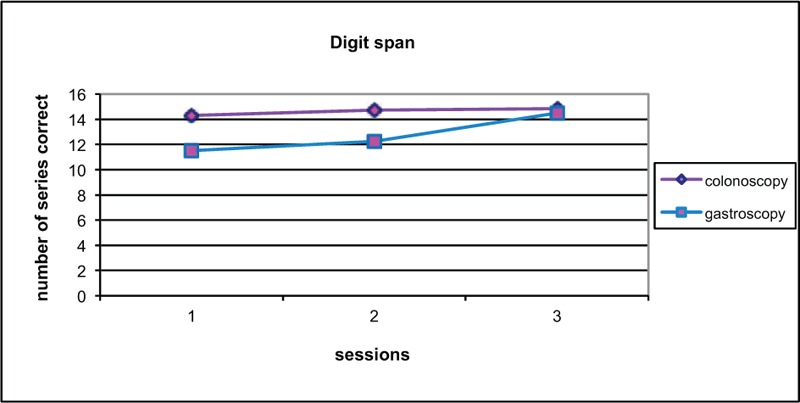
Overall digit span scores for colonoscopy and gastroscopy groups, respectively, were 14.3 and 11.5 (*P* = 0.36) at baseline, 14.7 and 12.3 (no significant difference *P* = 0.33) at test 2, 14.9 and 14.5 (no significant difference *P* = 0.92) at test 3.

Change in score from baseline to test 2 was compared (Figures 2–6). Both groups demonstrated improved performance in all tests with time. No significant difference between colonoscopy and gastroscopy groups was observed for any test.

Blood tests taken immediately before the procedure at the time of cannulation were compared. Potassium was significantly lower in the colonoscopy group at 3.8 versus 4.2 (*P* = 0.006) as was urea at 4.2 versus 5.9 (*P* =  < 0.0001). However, both remained within the normal range (Table 4).

**TABLE 4:**
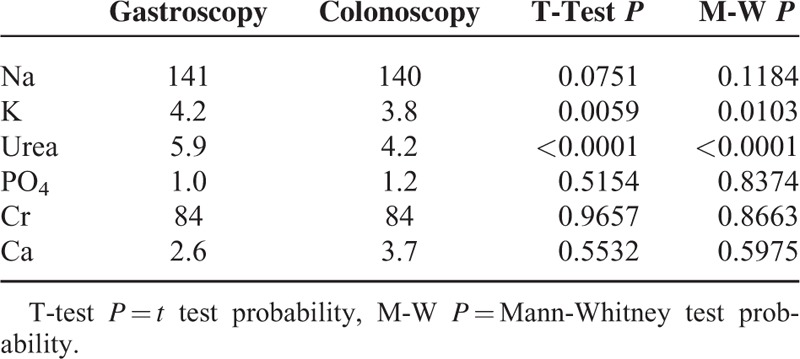
Results of Electrolyte Tests Taken at Time of Intervention

## DISCUSSION

This is the first study to evaluate the effect of bowel preparation and cognitive function; the results show that there is no evidence of cognitive impairment resulting from administration of bowel preparation before colonoscopy. The significance of this is that it confirms bowel preparation for colonoscopy will not directly impair a patient's capacity to consent to the procedure or affect their judgment regarding other important decisions. It does not indicate the precautions that are applied following sedation should also be applied before colonoscopy during the period of bowel preparation.

Groups in our study were matched for age and gender; however, the gastroscopy group were significantly heavier on average. There was no statistically significant difference in performance at baseline testing which demonstrates the groups were matched for baseline cognitive function. Colonoscopy and gastroscopy groups also demonstrated no significant difference in subsequent tests, which shows that cognitive function remained comparable to control following bowel preparation. Furthermore, there was no significant difference in change of score from baseline to test 2; therefore, patients in the colonoscopy group demonstrated the same ability to improve their performance with practice as the gastroscopy group. The tests were all conducted within a short time, around 1 week. This has the advantage of removing any significant possibility of developing a separate condition which could affect cognitive function. Although this did allow patients to become familiar with the tests with a resultant improvement with practice, this opportunity was the same for both groups therefore should not bias either group.

The CANTAB tests are a reliable validated tool in the assessment of a wide range of cognitive functions.^6,7^ Tests are computerized yet supervised by an administrator allowing reliable recording of results and correct performance by participants. They are sensitive enough to detect early subtle changes in cognitive function.^8^ The applications of the CANTAB tests are wide ranging and have previously been used to assess the cognitive effects of other drugs.^9^ The CANTAB test battery therefore provides an appropriate and reliable method of detecting whether bowel preparation has any effect on cognitive function.^8^

A similar but different study published in 2008 looked at the effect of dehydration with bowel preparation and cognitive function.^10^ They found that in the 38 patients that they studies that they lost on average 1.5 kg with the preparation; however, despite this degree of dehydration, all cognitive tests were within 1 SD of the population mean of normal values. This was quite a different study with an assumed population control, as opposed to this study which used the patient without bowel preparation and also another endoscopic procedure as a control.

The study is limited by size due to difficulties in recruitment. Shortly after the study commenced in 2010 a magnitude 7.1 earthquake hit causing significant damage within the city, and a large number of invitations to take part went unanswered. Following a further 6.3 magnitude earthquake in February 2011 the study was placed on hold due to disruption to the endoscopy service and displacement of a large number of people within the city. Further to this 1 person was unable to attend for colonoscopy due to heavy snow, and 3 patients’ results could not be included in the analysis due to computer errors. Patients with significant frailty (eg, those who required inpatient bowel preparation) or preexisting cognitive impairment were excluded therefore conclusions cannot be drawn regarding these groups. The results of the study may not be generalizable to all types of the bowel preparation, and further studies may be required of other preparation, however given the previous 2008^10^ study and this the results are likely to be similar.

This is the only clinical study so far to address the important clinical issue of bowel preparation and its effect on cognitive function, which could have significant implications for consent and the safety of the patient in the preprocedure period. This study did not find evidence of cognitive impairment resulting from administration of bowel preparation before colonoscopy. Further work could investigate whether bowel preparation before colonoscopy does affect cognition in higher risk groups.

## References

[1] Hsu YH Lin FS Yang CC Evident cognitive impairments in seemingly recovered patients after midazolam-based light sedation for diagnostic endoscopy. *J Formos Med Assoc* 2013; pii: S0929-6646(13) 00265-9. doi: 10.1016/j.jfma.2013.07.018. [Epub ahead of print]. 10.1016/j.jfma.2013.07.018 24035569

[2] Ip HY Chung F Escort accompanying discharge after ambulatory surgery: a necessity or a luxury? *Curr Opin Anaesthesiol* 2009; 22:748–754. 19745728 10.1097/ACO.0b013e328331d498

[3] Frizelle FA Colls BM Hyponatremia and seizures after bowel preparation: report of three cases. *Dis Colon Rectum* 2005; 48:393–396. 15812590 10.1007/s10350-004-0778-6

[4] Dillon CE Laher MS The rapid development of hyponatraemia and seizures in an elderly patient following sodium picosulfate/magnesium citrate (Picolax). *Age Aging* 2009; 38:487. 10.1093/ageing/afp054 19406975

[5] Folstein M Folstein S McHugh P “Mini mental state” a practical method for grading the cognitive state of patients for the clinician. *J Psychiatr Res* 1975; 12:189–198. 1202204 10.1016/0022-3956(75)90026-6

[6] Robbins TW James M Owen AM Cambridge Neuropsychological Test Automated Battery (CANTAB): a factor analytic study of a large sample of normal elderly volunteers. *Dementia* 1994; 5:266–281. 7951684 10.1159/000106735

[7] Lowe C Rabbitt P Test/re-test reliability of the CANTAB and ISPOCD neuropsychological batteries: theoretical and practical issues. *Neuropsychologia* 1998; 36:915–923. 9740364 10.1016/s0028-3932(98)00036-0

[8] Louis WJ Mander AG Dawson M Use of computerized neuropsychological tests (CANTAB) to assess cognitive effects of antihypertensive drugs in the elderly. Cambridge Neuropsychological Test Automated Battery. *J Hypertens* 1999; 17:1813–1819. 10703873 10.1097/00004872-199917121-00005

[9] Fray PJ Robbins TW Sahakian BJ Neuorpsychiatyric applications of CANTAB. *Int J Geriatr Psychiatry* 1996; 11:329–336.

[10] Ackland GL Harrington J Downie P Dehydration induced by bowel preparation in older adults does not result in cognitive dysfunction. *Anesth Analg* 2008; 106:924–929. 18292441 10.1213/ane.0b013e3181615247

